# The perception of peripersonal space in right and left brain damage hemiplegic patients

**DOI:** 10.3389/fnhum.2014.00003

**Published:** 2014-01-27

**Authors:** Angela Bartolo, Mauraine Carlier, Sabrina Hassaini, Yves Martin, Yann Coello

**Affiliations:** ^1^Laboratoire Unité de Recherche en Sciences Cognitives et Sciences Affectives, Université Lille Nord de FranceLille, France; ^2^Service de Neuropsychologie, Centre de rééducation et de réadaptations fonctionnelles spécialisées – Sainte Barbe, Fouquières les LensFrance; ^3^Service de Neuropsychologie, Centre de rééducation et de réadaptations fonctionnelles spécialisées – L’Espoir, HellemmesFrance

**Keywords:** perception and action, spatial vision, peripersonal space, brain damage, right left hemispheres

## Abstract

Peripersonal space, as opposed to extrapersonal space, is the space that contains reachable objects and in which multisensory and sensorimotor integration is enhanced. Thus, the perception of peripersonal space requires combining information on the spatial properties of the environment with information on the current capacity to act. In support of this, recent studies have provided converging evidences that perceiving objects in peripersonal space activates a neural network overlapping with that subtending voluntary motor action and motor imagery. Other studies have also underlined the dominant role of the right hemisphere (RH) in motor planning and of the left hemisphere (LH) in on-line motor guiding, respectively. In the present study, we investigated the effect of a right or left hemiplegia in the perception of peripersonal space. 16 hemiplegic patients with brain damage to the left (LH) or right (RH) hemisphere and eight matched healthy controls performed a color discrimination, a motor imagery and a reachability judgment task. Analyses of response times and accuracy revealed no variation among the three groups in the color discrimination task, suggesting the absence of any specific perceptual or decisional deficits in the patient groups. In contrast, the patient groups revealed longer response times in the motor imagery task when performed in reference to the hemiplegic arm (RH and LH) or to the healthy arm (RH). Moreover, RH group showed longer response times in the reachability judgment task, but only for stimuli located at the boundary of peripersonal space, which was furthermore significantly reduced in size. Considered together, these results confirm the crucial role of the motor system in motor imagery task and the perception of peripersonal space. They also revealed that RH damage has a more detrimental effect on reachability estimates, suggesting that motor planning processes contribute specifically to the perception of peripersonal space.

## INTRODUCTION

Spatial perception in relation to the body and the motor system has been the focus of extensive scientific investigations since the late 19th and early 20th centuries. This was observed in many different disciplines including philosophy (e.g., Bergson, 1896; Husserl, 1907/1998; Merleau-Ponty, 1945), mathematics (e.g., Poincaré, 1907) and slightly later in ethology and sociology (e.g., Hediger, 1934; Sommer, 1959; Hall, 1966), as well as in psychology and neurosciences (e.g., Brain, 1941; Gibson, 1979). The idea that has emerged from these studies is that locations in space are not to be defined as objective positions in relation to the objective position of our body; rather they inscribe around us the variety of reaches that our body and limbs can produce. For a cognitive system, spatial representation depends thus essentially on past experiences about opportunities, effects and costs of acting in a given environment (Previc, 1998; Proffitt, 2006). Recently, the representation of peripersonal and extrapersonal space has been widely investigated (Berti and Rizzolatti, 2002; Holmes and Spence, 2004; Coello and Delevoye-Turrell, 2007; Gallese, 2007; Witt and Proffitt, 2008; Caggiano et al., 2009). The ability to perceptually delimitate our peripersonal space is critical since peripersonal space contains the objects that we can immediately reach for, specifies our private area in social interactions and contains the obstacles to which the organism must pay attention in order to avoid colliding with them, particularly when gesturing. In the past, several studies have shown that people are quite accurate in visually delimiting their peripersonal space when evaluated through reachable estimates (e.g., Carello et al., 1989; Fischer, 2000; Coello and Iwanow, 2006; Gabbard et al., 2006), although the latter have been found to be influenced by the environmental context (Coello and Iwanow, 2006), the emotional state (Kennedy et al., 2009), the postural constraints (Rochat and Wraga, 1997; Fischer, 2000; Gabbard et al., 2007), and even the presence of mental or neurological illness (Coello and Delevoye-Turrell, 2007; Delevoye-Turrell et al., 2011).

As peripersonal space is structured by action, it has been suggested that the perception of objects in peripersonal space requires a motor-based perceptual system combining visual with motor- and body-related variables (Coello and Delevoye-Turrell, 2007; Gallese, 2007; Witt and Proffitt, 2008). Evidence for the contribution of motor-related information in the perception of peripersonal space was mainly supported by the observation that the motor neural network is involved in the perception of near objects (Grezes et al., 2003; Caggiano et al., 2009; Gallivan et al., 2009; Rossit et al., 2013) but also reachability judgments (Bartolo et al., 2009) that includes brain areas that overlap with those recruited for actual motor planning and execution. Furthermore, the involvement of brain motor areas in the perception of peripersonal space has been supported by the effect of depressing cortical excitability of the motor cortex while performing a reachability judgment task. By using transcranial magnetic stimulation (TMS) at low frequency, Coello et al. (2008) revealed an interference effect in a reachability judgment task performed in reference to the right arm when TMS was applied over the left motor cortex, which was not observed when TMS was applied over the left temporo-occipital area stimulated as a control site. The contribution of the motor system to the perception of peripersonal space is assumed to involve predictive models based on action simulation and anticipation of action-related sensory effects (Jeannerod, 2006; Gallese, 2007). Hence, action feasibility may be evaluated and actual action guidance can be facilitated (Jeannerod, 2001). Accordingly, individuals with brain lesions affecting actual motor performances or motor imagery are expected to be also impaired in the perception of peripersonal space, which has not been thoroughly investigated so far.

Complementarily, studies in stroke patients have revealed that motor impairments following brain damage have different characteristics depending on whether the brain lesion is located within the right or the left brain hemisphere (Schaefer et al., 2007, 2009b). Indeed, although arm control for reach and prehension arises primarily from descending projections originating from the contralateral cortex and brainstem (Kuypers, 1982), more recent electrophysiological and brain imaging studies have also shown substantial activation of ipsilateral motor cortex during unilateral hand and arm movements, suggesting a role of both hemispheres in controlling the dominant limb (Kutas and Donchin, 1974; Tanji et al., 1988; Kim et al., 1993; Kawashima et al., 1994; Gitelman et al., 1996). Assuming that each hemisphere is specialized for controlling different aspects of voluntary actions, unilateral brain damage to the left and right hemisphere (RH) was expected to result in distinct deficits depending on the side of the lesion with more specifically abnormalities in the control of the ipsilesional arm (Winstein and Pohl, 1995; Desrosiers et al., 1996; Carey et al., 1998; Yarosh et al., 2004; Wetter et al., 2005). Indeed, lesions in the hemisphere controlling the dominant arm was found to mainly produce deficits in the spatio-temporal features of motor trajectories, suggesting a deficit in the on-line control of voluntary action (Haaland and Delaney, 1981; Haaland and Harrington, 1996; Prestopnik et al., 2003). Contrasting with this result, stroke patients with lesions in the hemisphere controlling the non-dominant arm was found to mainly produce deficits in final position accuracy of the dominant arm, suggesting a specific deficit in the accurate planning of the initial parameters of voluntary action, with no impairment in on-line control (Haaland and Delaney, 1981; Winstein and Pohl, 1995; Haaland and Harrington, 1996; Prestopnik et al., 2003). Consistent with this, recent studies compared right-handed patients with age-matched controls in a manual-reaching task and reported that patients with left hemisphere (LH) lesions were characterized by specific decreases in movement speed and increases in trajectory curvature, with also lower smoothness (Winstein and Pohl, 1995; Schaefer et al., 2007, 2009a,b; Haaland et al., 2009). These findings strongly suggest that the LH plays an important role in the integration of visual and motor information during the execution of voluntary motor action and the control of complex motor skills (Gonzalez et al., 2006, 2007; Ketcham et al., 2007). By contrast, right-handed patients with RH lesions showed larger reaction times and increased final position errors (Winstein and Pohl, 1995; Schaefer et al., 2007, 2009b), suggesting a specific deficit in the early motor planning and/or programing processes of accurate actions (Ketcham et al., 2007). In support of this, right hemisphere damaged patients show more spatio-temporal deficits when performing a voluntary action in an open loop than in a closed loop condition, the latter offering more opportunities for visual on-line correction (Rossit et al., 2009). They also show specific deficits when requested to plan an action according to predefined cognitive constraints (e.g., off-line compared to on-line action control – Rossit et al., 2011). Moreover, right-handed patients with RH lesions also showed longer response times in motor imagery tasks, with less temporal congruency between temporal aspects of real and imagined movements (Stinear et al., 2007; Malouin et al., 2008; Vromen et al., 2011; Malouin et al., 2012). Thus, the difficulty in planning a voluntary action seems to correlate with the difficulty in imagining the same action (Dominey et al., 1995; Sirigu et al., 1995).

In the present study, we analyzed the perceptual performance of right-handed hemiplegic patients with brain damage to the left or to the right hemisphere, in three tasks: a color discrimination, a motor imagery, and a reachability judgment task. In relation with the literature reported above suggesting that the planning of a voluntary action relies predominantly on the RH, we expected hemiplegic patients with brain damage localized to the RH to be more impaired in tasks involving motor representations, i.e., the motor imagery and the reachability judgment tasks.

## MATERIALS AND METHODS

### PARTICIPANTS

The twenty-four participants involved in this study comprised eight patients (four males) with hemiplegia due to lesions to the LH (mean age: 50 years, SD: 16.91 years; mean arm length: 74.53 cm, SD: 7.41 cm), eight patients (seven males) with hemiplegia due to lesions to the RH (mean age: 52.88 years, SD: 14.17 years; mean arm length: 76.84 cm, SD: 5.22 cm), and eight healthy controls (HC, seven males, mean age: 48.75 years, SD: 15.08 years; mean arm length: 74.44 cm, SD: 5.08 cm). The study was performed in agreement with the local ethical committee guidelines and in accordance with the principles of Helsinki declaration. All patients and HCs gave written formal consent before being included in the study. All participants were right-handed, as ascertained by the handedness Inventory Scale (Oldfield, 1971).

Patients were selected such that they exhibited hemiplegia contralateral to the lesion side, i.e., a severe or complete loss of motor function of the arm, but with a normal or corrected to normal vision. Patients with hemineglect (Rousseaux et al., 2002), or with previous episodes of neurological or psychiatric disorders were excluded from the study. Before being involved in the study, patients were administered a series of neuropsychological tasks. Furthermore, force-stretching capability for both arms was also evaluated. The complete demographic data for the HCs as a group and for each individual patient appear in **Table 1**. There were no significant differences among the three groups concerning the age (*F*_2,23_ = 0.15, *p* = 0.86) and the level of education (*F*_2,23_ = 0.39, *p* = 0.68).

**Table 1:** Demographical data of healthy controls (HC) as well as LH and RH patients.

Participant	Group	Age	Education	Gender (M/F)	Lesion
Healthy controls	HC	Mean: 48.75 SD: 15.08	Mean: 12.4 SD: 4.2	7/1	None
L1	LH	45	17	F	Ischemic sylvian stroke
L2	LH	39	12	M	Haematoma insulo-lenticular
L3	LH	82	8	F	Ischemic sylvian stroke
L4	LH	25	8	M	Traumatic head injury
L5	LH	47	8	M	Ischemic left dorsolateral pontine stroke, cerebellum, lenticular nucleus
L6	LH	49	8	F	Ischemic sylvian stroke, lenticular nucleus, internal capsule, caudate nucleus
L7	LH	49	17	M	Hemorrhagic stroke
L8	LH	64	8	F	Ischemic stroke
R1	RH	31	8	M	Traumatic head injury
R2	RH	45	8	M	Ischemic sylvian stroke
R3	RH	51	12	M	Ischemic sylvian stroke
R4	RH	52	8	M	Traumatic head injury
R5	RH	52	12	M	Ischemic sylvian stroke
R6	RH	54	12	M	Ischemic stroke
R7	RH	56	12	M	Hemorrhagic stroke, hematoma capsulo- lenticular
R8	RH	82	17	F	Ischemic sylvian stroke

*LH, left hemisphere damage; RH, right hemisphere damage; M, male; F, female.*

### PATIENTS ETIOLOGY

Patients were recruited and tested at the Centre of Neuropsychology Sainte Barbe and at the Centre L’Espoir, both located in Lille neighborhoods. Of the eight LH patients, six had suffered from strokes, one had a traumatic head injury, and one had haematoma. Of the eight RH patients, six had suffered from strokes, and two had a traumatic head injury. At the time of testing, all patients were hemiplegics for the side contralateral to the lesion. Patients were tested between two weeks to three months after pathology onset.

### NEUROPSYCHOLOGICAL PROFILE

#### Controls

All HCs had a score above the cut-off at the MATTIS Dementia Rating Scale (Mattis, 1973, adapted in French, GRECO, 1994; range: 139–144; mean: 142.8, SD: 1.9).

#### Patients

The tests administered to patients consisted in a neuropsychological evaluation: (1) *executive functions* were assessed using the Batterie Rapide d’Efficience Frontale (BREF) test (Dubois et al., 2000); (2) *attention* was assessed using the Test d’Evaluation de l’Attention (TEA) test (Zimmermann and Fimm, 1993); (3) *visuo*-*spatial abilities*: visual memory was assessed with the Door test (Baddeley et al., 1994) and short term spatial memory was assessed using the Corsi block tapping test (Corsi, 1972); and (4) *verbal abilities*: general verbal abilities were evaluated using the naming test (Bachy-Langedock, 1989), verbal comprehension using the MT86 task (Nespoulous et al., 1992), and the short-term memory using the forward and backward digit span tasks (Wechsler, 1991). Results obtained in the neuropsychological tests achieved by the two patient groups are reported in **Table 2**. Statistical comparisons between the two patients group were run using *t*-test for independent groups. For all comparisons, equal variance was found (Levene’s test; *p* > 0.1 in all cases). For the TEA test, a series of frequency analyses (Chi-square) were run to check for differences between the LH and RH patients who passed the different sub-tests. Overall, results showed an absence of difference between the two groups of patients on all the evaluation tests, except for the evaluation of phasic alertness in which patients with damage to the RH showed slower responses than patients with damage to the LH. This result is in line with previous studies showing that phasic alertness is usually affected in right brain damage patients (Robertson et al., 1998; Longoni et al., 2000). These differences however, did not appear when a warning signal was previously provided. Furthermore, anosognosia was not found in any patient, when tested using Della Sala et al.’s (2009) test.

**Table 2:** Cognitive and physical scores for the two patients’ group (the number of participants included in the analysis varied depending on the test due to failure of the evaluation in some cases).

		LH		RH	Statistics
	Scores	Number of patients above cut-off/total number of patients	Scores	Number of patients above cut-off/total number of patients	
**Executive functions**
BREF^1^	13.88 (2.1)	4/8	13.38 (2.7)	2/8	*t*_14_ = 0.41, *p* = 0.69
**Attention**
TEA^2^					
Divided attention					
Auditory		5/7		4/7	χ^2^_1; 0.95_ = 1.77, *p* = 0.18
Visual		5/7		3/7	χ^2^_1; 0.95_ = 2.97, *p* = 0.08
Go no-go		6/8		6/7	χ^2^_1; 0.95_ = 0.64, *p* = 0.42
Phasic alertness					
With warning signal		4/7		4/8	χ^2^_1; 0.95_ = 3.36, *p* = 0.06
Without warning signal		5/7		2/8	χ^2^_1; 0.95_ = 13.48, *p* < 0.01
**Visuo-spatial abilities**
Door	12.75 (5.12)	5/8	10.13 (4)	3/8	*t*_14_ = 1.14, *p* = 0.27
Corsi	4.29 (1.4)	8/8	4.25 (0.9)	8/8	*t*_13_ = 0.06, *p* = 0.95
**Verbal abilities**
Naming	79.88 (7.1)	4/8	83.5 (5.58)	6/8	*t*_14_ = -1.13, *p* = 0.28
Comprehension MT86	44.71 (1.9)	7/8	45.40 (1.9)	7/8	*t*_10_ = -0.60, *p* = 0.56
Digit span forward	4.25 (1.6)	7/8	4 (1)	7/8	*t*_13_ = 0.36, *p* = 0.73
Digit span backward	3 (1.07)	5/8	3.29 (0.76)	7/8	*t*_13_ = -0.59, *p* = 0.57
**Physical attitude**
Force healthy arm	39 kg (15.4 kg)		42.2 kg (9.1 kg)		*t*_13_ = -0.49, *p* = 0.64
Force hemiplegic arm	10.6 kg (12.1 kg)		7 kg (10.5 kg)		*t*_13_ = 0.61, *p* = 0.55
Stretching healthy arm	180°		180°		ns
Stretching hemiplegic arm	34° (41°)		24° (42°)		*t*_14_ = 0.46, *p* = 0.65

*Notes: ^1^Dubois et al. (2000), Cut-off equal 15; ^2^Scores were not available for this task, therefore, only the number of individuals above cut-off has been reported.*

### PHYSICAL ASSESSMENT

Given the nature of our tasks, the force and the amplitude of action performed with both the healthy and hemiplegic arm were evaluated in the patient groups. The force (in kg) was measured with the Jamar hydraulic hand dynamometer; for the arm stretching amplitude, the angular displacement of the shoulder was measured with a goniometer. Statistical comparisons were run using *t*-test for independent groups assuming equal inter-group variances (Levene’s test, all *p* > 0.1). Concerning the patients’ healthy arm, no differences were registered for force (mean score LH: 39.0 kg, SD = 15.4 kg; RH = 42.2 kg, SD = 9.1 kg; *t*_13_ = -0.49, *p* = 0.64) and stretching capacities (mean score: 180°, ns) between the two patient groups. Concerning the hemiplegic arm, we also found that both the force (mean score LH: 10.6 kg, SD = 12.1 kg; RH: 7 kg, SD = 10.5 kg; *t*_13_ = 0.61, *p* = 0.55) and the arm stretching capacities (mean score LH: 34°, SD = 41°; RH: 24°, SD = 42°; *t*_14_ = 0.46, *p* = 0.65) did not differ between the two patient groups. Given the lack of significant differences between the two patient groups as far as the healthy and the hemiplegic arms were concerned, the measure of force and arm stretching for the healthy and the pathological arms were collapsed across individuals, respectively. Overall, the hemiplegic arm was more affected than the healthy arm for both the force (*t*_14_ = 9.68, *p* < 0.001) and the stretching amplitude (*t*_15_ = 14.89, *p* < 0.001).

### MATERIALS AND PROCEDURES

All participants were administered three tasks: a color discrimination, a reachability judgment, and a motor imagery task described hereafter.

#### Color discrimination and reachability judgment task

**FIGURE 1:**
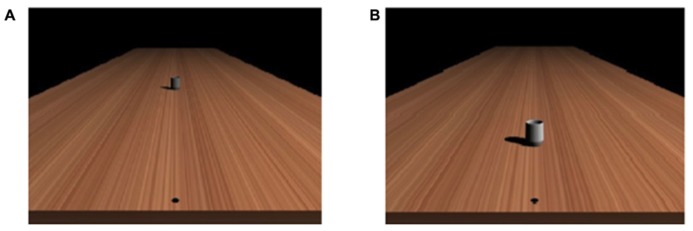
**Examples of the stimuli used in (A) the reachability judgment task and (B) the color discrimination task**.

For both the color discrimination and the reachability judgment task, the experiment was run on a computer using E-prime program (Psychology Software Tools, Inc. www.pstnet.com). Event stimuli consisted in the presentation of a picture created with a 3D graphics software (Blender 3D modeler under GNU General Public License) and represented a virtual scene with a mug (height: 7.5 cm, diameter: 4.2 cm) lying on a table (see **Figure 1**). The geometry of the visual scene was computed with a vantage point at eye level 43 cm above the horizontal surface. The virtual surface on which the objects were presented was a 2 m × 8 m rectangular surface made with a homogenous texture. On the surface, a black dot was displayed 5 cm from the nearest side of the table on the sagittal axis. Participants were instructed to imagine having their right index finger on the black dot while performing the perceptual task. The surface of the table was generated with a linear texture extracted from a picture of a piece of wood, which produced a realistic rendering using a ray-tracing algorithm with shadow calculation, but with no information about absolute distance (see Morley and Morley, 2003). Due to the geometry of the virtual scene, the distance of the visible object could be estimated mainly on the basis of the relative size and perspective cues.

In the color discrimination task, the stimulus was a mug, placed consistently at 50 cm from a starting location and with a color that could vary from bright to dark. The different colors of the mug were selected from a preliminary study that tested the value that individuals judged on average as located in between bright and dark color, and the range of the colors used in the present study corresponded to ± 2 SD according to this average value. The color of the mug (considering the average value between red, green, and blue channels using the 0 to 255 RGB levels scale) was thus comprised between bright (103.09) and dark (29.26), by step of 2.546, providing thus 30 possible colors for the mug.

In the reachability judgment task, the same mug (averaged value across color channels: 66.33) was placed in perspective at different distances with respect to the starting location (from 30 to 175 cm by step of 5 cm, providing thus 30 possible distances, see **Figure 1**).

The color discrimination and the reachability judgment tasks were run in two separate sessions. E-prime software was used to control stimuli delivery and to record participants’ responses. To get participants acquainted with the tasks, they were provided with a few practice trials of each task before starting the experimental session. At the beginning of each task, participants were provided with written instructions presented on the screen about the task they had to perform. In each condition, the task instruction disappeared from the screen when the participant pressed on the space bar of the computer keyboard. Then, the first stimulus appeared on the screen and remained visible until the participant’s response was provided, within a time window of maximum 4 s followed by another 2 s of black screen. The other stimuli were presented randomly according to the same temporal sequence.

In the color discrimination task, participants were instructed to indicate whether the mug (at constant location) on the screen had a bright or dark color. In the reachability judgment task, participants were required to estimate if the mug (of similar color) was reachable or unreachable. To make their answer as simple as possible, at the beginning of each task the instructions were provided in the form of a simple verbal question: Is the stimulus bright? or is the stimulus reachable? This offered two possible answers for the participants: yes or no. Responses were provided verbally; hence, while the quality of the response (bright/dark, reachable/unreachable) was registered by the examiner, verbal response times were collected using SR-BOX of E-prime software, which was equipped with a microphone. In each task, each stimulus was presented five times. Participants performed then 150 trials in each task, which lasted about 12–15 min leading to a total duration of each session of about 36–45 min (3 tasks × 12–15 min). The color discrimination task served as a control task to evaluate whether the patients showed specific impairment in processing visual information or selecting the appropriate motor response (with the healthy hand). It is worth noting that the color discrimination task contains a region of ambiguity according to the decision to make (transition area between bright and dark decision), as it is the case for the reachability judgment task (transition area between reachable and unreachable stimuli; Coello et al., 2008). Therefore, the two tasks had similar difficulties in relation to the decision process.

#### Motor imagery task

For the motor imagery task, we used a finger-to-thumb opposition task. This consisted in the participants to oppose the fingers of the hand to the thumb following the finger order (index, middle, ring, and little finger). The sequence was repeated five times at a self-imposed pace in two conditions:

(1) Actual motor condition: the experimenter gave a go signal and recorded the time between this signal and the actual completion of the five movement cycles.

(2) Imagined motor condition: the experimenter gave the go signal and recorded the time between this signal and the verbal indication from the participants when they indicated having accomplished the five imagined motor sequences. Participants were instructed to concentrate on the quality of the performance rather than the speed of the movement.

All participants completed the imagined motor condition with both hands, thus with the intact and hemiplegic hand for the patients. However, the actual motor task was executed by the patients with their healthy hand only, while the HCs performed the task with both hands. Previous studies have shown that the time to imagine making a movement and the time to actually execute the same movement are very similar in healthy participants (e.g., Decety et al., 1989), suggesting the existence of common underlying cognitive and neural mechanisms (Decety, 1996; Jeannerod, 2001). For each finger-to-thumb opposition task, response times were registered by means of a chronometer.

### DATA ANALYSIS

In the color discrimination task and the reachability judgment task, the transition between one type of response (reachable-bright) to the other (unreachable-dark) was computed using a maximum likelihood fit procedure based on the second-order derivatives (Quasi-Newton method) to obtain the logit regression model that best fitted the reachable (bright)/unreachable (dark) responses of the participants, using the equation: y=e^(α +βX)^ / (1+e^(α +βX)^, in which y was the participant’s response, X corresponded to the distance, (-α/β) was the critical value of X at which the transition from one type of response (reachable-bright) to the other type of response (unreachable-dark) occurred, thus expressing the perceived maximum reachable distance or the color perceived mid-way between brightness and darkness. Response times were also analyzed, but differentiating response times for reachable (bright) and unreachable (dark) stimuli, as well as for stimuli at the boundary of reachable space or at the threshold between bright and dark color (corresponding hereafter to the Distance and Brightness factors).

In the motor imagery task, data analysis was performed on the average scores computed for each participant from the response times obtained in each of the five actual and imagined motor sequences. For the reachability judgment task and the color discrimination task, the participants’ perceptual thresholds and movement times were analyzed for the healthy and hemiplegic hands. Statistical analyses were performed on the participants’ scores obtained in the different tasks by means of analyses of variances (ANOVAs) computed with Statistica software. Kolmogorov–Smirnov test was used to check for normality of data distribution in each participants group. Homogeneity of variance was estimated using Levene’s test and non-parametric analyses were used when the assumptions of homogeneity of variance or normality were violated (*p* < 0.1). In these cases, the information on the variance homogeneity, or non-normality of distribution, was added in the core of the text. *Post hoc* analyses were performed using standard Tukey procedures with α correction for multiple comparisons.

## RESULTS

All data were normally distributed (Kolmogorov–Smirnov test, *p* > 0.05), therefore we used parametric analysis of variance for testing for main factors and interactions effects. When between-group variances were unequal (Levene’s test, *p* < 0.10), non-parametric analysis was performed.

### COLOR DISCRIMINATION TASK

#### Boundary of bright-dark discrimination

A one-way ANOVA (Group) on the color threshold differentiating bright from dark stimuli did not show any significant difference among the three groups (mean values for HC: 60.19, SD: 6.85; LH: 61.80, SD: 11.01; RH: 60.19; SD: 6.85; Welch’s test: *F*_2,12.54_ = 0.19, *p* = 0.83, to account for unequal variance with Levene’s test: *p* = 0.057).

#### Response times

**FIGURE 2:**
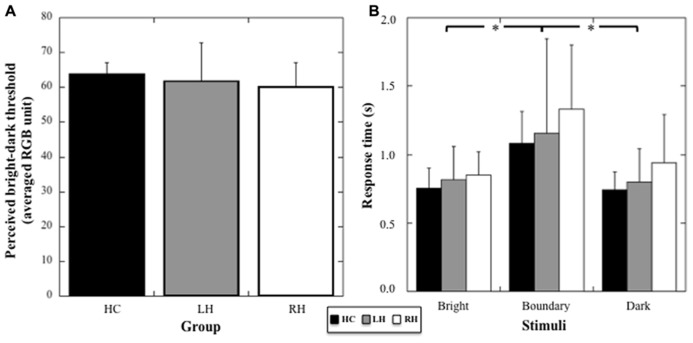
**(A)** Mean threshold (and SD, in averaged RGB unit) in the color discrimination task for HC, RH, and LH groups. **(B)** Mean response times (and SD, in seconds) for the stimuli judged bright, dark, or at the boundary between bright and dark color. Stars indicate significant statistical difference.

A two way-ANOVA (Group × Brightness) on response times showed no effect of the Group (*F*_1,21_ = 2.76; *p* = 0.19), but a significant effect of Brightness (*F*_2,42_ = 19.88, *p* < 0.001, η^2^ = 0.49), with no interaction between the two factors (*F*_4,42_ = 0.64, *p* = 0.63, see **Figure 2**). *Post hoc* comparisons showed a significant increase in response times for stimuli at the threshold compared to bright and dark stimuli (*p* < 0.001 for both comparisons). No difference between stimuli perceived as bright and dark was registered (*p* = 0.71). Thus, the data suggest an absence of any specific deficit in visual processing or decision taking in the patient groups.

### REACHABILITY JUDGMENT TASK

#### Boundary of reachable-non-reachable discrimination

**FIGURE 3:**
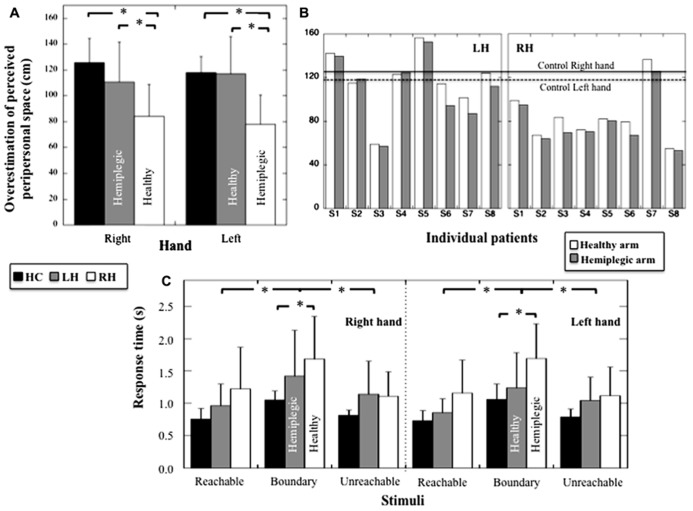
**(A)** Mean overestimation (and SD, in cm) of perceived peripersonal space for HC, LH, and RH for judgments provided according to the right or the left hand. **(B)** Individual overestimation (in cm) of perceived peripersonal obtained with the hemiplegic and healthy hand for LH and RH. Mean performances obtained with the right and left hand for HC are also indicated. **(C)** Mean response times (and SD, in seconds) for the stimuli judged reachable, unreachable, or at the boundary or reachable space for HC, LH, and RH and for judgments provided according to the right or the left hand. Stars indicate significant statistical difference.

Because peripersonal space perception was different when evaluated from reachability judgment relative to the right or left hand in HC (i.e., perceived reachable space decreased when assessed relative to the left hand, *t*_7_ = 2.72, *p* = 0.03), we performed a two-way ANOVA including the Group (HC, RH, LH) and the Hand (left, right) factors (see **Figures 3A** and **3B**). We found a significant effect of Group on reachability judgments (*F*_2,21_ = 6.605, *p* = 0.006; η^2^ = 0.39). *Post hoc* analyses (Tukey test) showed that peripersonal space decreased in RH group compared to HC (*p* = 0.007) and LH group (*p* = 0.012); no difference between HC and LH was present (*p* = 0.51). No significant effect of the Hand used was found (*F*_1,21_ = 2.99, *p* = 0.09), but a significant Group × Hand interaction was registered (*F*_2,21_ = 8.5, *p* = 0.002, η^2^ = 0.45). *Post hoc* comparisons revealed a decrease of perceived peripersonal space when estimated according to both the left (pathological) and right (healthy) hand in RH compared to HC (*p* = 0.023 and *p* = 0.018 respectively). Furthermore, a decrease in perceived peripersonal space was observed in RH compared to LH for the pathological hand (*p* = 0.018) and the healthy hand (*p* = 0.038). Moreover, RH and LH groups showed that the decrease of peripersonal space was more pronounced when reachability judgments were provided relative to the pathological hand compared to the healthy hand (*p* = 0.026 and *p* = 0.028, respectively). Thus, RH patients revealed a decrease of perceived peripersonal space when estimated according to both the healthy and hemiplegic arm, whereas LH patients revealed a decrease of perceived peripersonal space when estimated according to the hemiplegic arm only.

#### Response times

A three way-ANOVA (Group × Hand × Distance) on response times showed an absence of Group effect (*F*_2,21_ = 3.07; *p* = 0.06), no effect of the hand (*F*_1,21_ = 1.79, *p* = 0.19), no Group × Hand interaction (*F*_2,21_ = 0.85, *p* = 0.44), no Hand × Distance interaction (*F*_2,42_ = 0.087, *p* = 0.92) and no Hand × Distance × Group interaction (*F*_4,42_ = 0.37; *p* = 0.83) on response times (see **Figure 3C**). There was however, an effect of Distance (*F*_2,42_ = 39.64, *p* < 0.001, η^2^= 0.65), and an interaction between Group × Distance (*F*_4,42_ = 2.73, *p* = 0.042; η^2^ = 0.21). *Post hoc* comparisons (Tukey test) showed a significant increase in response times for stimuli located at the boundary of reachable space in RH compared to HC (*p* = 0.041) but an absence of difference when comparing LH and HC (*p* = 0.64). No difference was observed among RH, HC, and LH when considering reachable (*p* = 0.30 and *p* = 0.6 respectively) and unreachable stimuli (*p* = 0.53 and *p* = 0.9 respectively). Thus, only the RH patients revealed impaired response times when estimating stimuli located at the boundary of peripersonal space.

### MOTOR IMAGERY TASK

#### Actual vs. imagined actions

**FIGURE 4:**
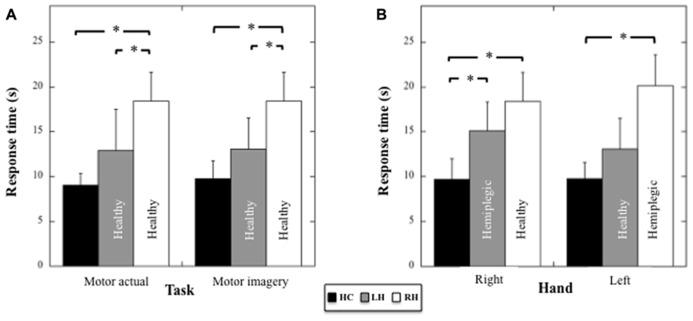
**(A)** Mean response times (and SD, in seconds) for HC, LH, and RH in the motor actual and imagery tasks performed with the healthy hand. **(B)** Mean response times (and SD, in seconds) for HC, LH, and RH in the motor imagery tasks performed with the right or the left hand. Asterisk indicate significant statistical difference.

Because the patients used a different hand in the actual motor task, we compared the performances between patients and controls in the actual and in the motor imagery task (healthy hand only) by using for the actual and motor imagery task in HC the averaged performance (mean = 9.01 s, sd = 1.35 s and mean = 9.75 s, sd = 2.01 s, respectively) between the right (mean = 8.89 s, sd = 1.62 s and mean = 9.7 s, sd = 2.3 s, respectively) and the left hand (mean = 9.12 s; sd = 1.10 s and mean = 9.8 s, sd = 1.8 s, respectively), for which the performance did not differ statistically (respectively *t*_7_ = 0.53, *p* = 0.61 and *t*_7_ = 0.19, p = 0.85). A two-way ANOVA (Group × Task) on response times (see **Figure 4A**) revealed an effect of the Group (*F*_2,17_ = 16.5; *p* < 0.001; η^2^ = 0.66), no effect of the Task (*F*_1,17_ = 0.25; *p* = 0.6), and no Group × Task interaction (*F*_2,17_ = 0.14; *p* = 0.9). *Post hoc* comparisons (Tukey test) revealed faster response times in HC compared to RH (*p* < 0.001) and in RH compared to LH (*p* = 0.009), but no difference, although close to significance, between HC and LH (*p* = 0.06). Hence, the RH patients showed a specific slowness in producing both actual and imagined actions with the healthy hand.

#### Motor imagery performances

Motor imagery performances in the three groups as a function of the Hand (healthy versus pathological hand for the patients) were analyzed using a two-way ANOVA (Group × Hand) on response times (see **Figure 4B**). Results showed a main effect of the Group (*F*_2,17_ = 20.09, *p* < 0.001, η^2^ = 0.70), an absence of effect of the Hand (*F*_1,17_ = 4.36, *p* = 0.86), and a significant Group × Hand interaction (*F*_2,17_ = 4.36, *p* = 0.03, η^2^ = 0.34). With respect to the right hand, *post hoc* comparisons (Tukey test) revealed faster response times for HC compared to RH (healthy hand, *p* < 0.001) and LH (pathological hand, *p* = 0.016), but an absence of differences between RH and LH (*p* = 0.39). With respect to the left hand, *post hoc* comparisons showed faster response times for HC compared to RH (pathological hand, *p* < 0.001), but not when compared to LH (healthy hand, *p* = 0.28). A significant difference between RH and LH persisted (*p* = 0.004). Thus, motor imagery was slower for both hands in RH, but only for the contralesional hand in LH.

## DISCUSSION

The aim of the present study was to evaluate the consequence of right or left hemiplegia in a group of patients affected by neurological damage to the left (LH) or RH, on perceived peripersonal space and in relation to motor imagery capabilities. To this end, a motor imagery and a reachability judgment task was employed. The latter task is known to depend on how individuals represent their peripersonal space (Coello and Delevoye-Turrell, 2007; Bourgeois and Coello, 2012). In the reachability judgment task, individuals are usually asked to judge whether a visual stimulus presented along the mid-sagittal axis at different distances is reachable or not according to their own action capabilities. Since no real action is performed, to provide an estimation of reachability decision mechanisms are thought to involve motor representations, as suggested by previous studies (Coello et al., 2008; Bartolo et al., 2009). Finally, to control for perceptual deficits and difficulty in responding, a control task was used which consisted in judging the brightness of a visual stimulus. This task had the same decisional constraints as the reachability judgment task since participants must classify visual stimuli in two categories with an area of decisional uncertainty when the presented stimuli were close to the perceptual threshold.

Results showed that for the color discrimination task, RH and LH patients performed the task similarly as the group of matched HCs. In particular, we found that the color corresponding to the threshold separating bright and dark stimuli (60.72 on average on the RGB color scale) was not affected by the cognitive impairments resulting from the brain insult. Furthermore, we found that response times were dependent on whether the stimuli presented were clearly bright, dark, or at the perceptual threshold of the bright-dark distinction. In particular, response times were equivalent for bright and dark stimuli, but increased significantly for the stimuli located near the threshold, due obviously to the ambiguity of those stimuli, which affected the decisional process. This pattern of results was identical across groups (LH, RH, and HC), which suggested that brain damage in our patients impaired neither the visual nor the decisional processes.

Concerning the reachability judgment task, overall we found that participants overestimated their actual peripersonal space. Indeed, while arm length was on average 75.27 cm, participants, considered the boundary of perceived peripersonal space as located at about 105 cm, which corresponded to an overestimation of about 71%. This overestimation is in agreement with some previous studies (see Delevoye-Turrell et al., 2010 for a thorough discussion), even if the value obtained in the present study was above that usually reported, which is around 10% (Carello et al., 1989; Coello and Iwanow, 2006; Coello and Delevoye-Turrell, 2007). Because we used a virtual scene, widely impoverished in terms of distance cues, one may assume that participants assessed the stimulus distance referring predominantly to familiar size of the visual stimuli (Treisilian et al., 1999). If that were the case, the over estimation of peripersonal space could result from the fact that participants perceived the mug much smaller than its actual size in the virtual scene, with the consequence that they will perceive it closer and thus, more reachable. Assuming a usual 10% of overestimation as indicated in the literature (see Delevoye-Turrell et al., 2010), this indicates that the perceived size of the mug should be about 30% smaller than its actual size, which corresponds to a mug with a height of 5.21 cm and a width of 2.91 cm. This might perhaps correspond to a more realistic mug. Regardless of its magnitude, overestimation has been attributed in the past to a biased representation of postural constraints (the *postural stability hypothesis*, Robinovitch, 1998; Gabbard et al., 2009), the preconceive potential actions based on multiple degrees of freedom instead of the one degree of freedom imposed by the task (the *whole body engagement theory*, Carello et al., 1989; Mark et al., 1997; Rochat and Wraga, 1997; Fischer, 2000; Gabbard et al., 2007). It could also be due to a high state of confidence about current motor possibilities (the *cognitive state hypothesis*, Gabbard et al., 2006).

Patients with a RH insult leading to left hemiplegia revealed however, a slightly different pattern of results compared to the two other groups. Indeed, patients in RH group showed a much reduced perception of the peripersonal space, with a reduction that reached 31% compared to that measured in the two other groups, whether the estimates were provided in reference to the healthy or hemiplegic arm. This indicates that a brain damage in the RH resulting in left hemiplegia affected widely the perception of peripersonal space. This interpretation was corroborated by the observation in RH group that response times increased significantly with respect to HC performance for stimuli located at the boundary of perceived peripersonal space. Interestingly, Coello et al. (2008) found the same pattern of results when transiently inhibiting the motor cortex using TMS in healthy participants performing a reachability judgment task. However, in the present study this pattern of result was not observed in LH group. The fact that the same effect was observed when the judgments were performed in reference to both the healthy and the hemiplegic arm indicates that the lower performances observed in the RH group was not the mere consequence of hemiplegia resulting from lesions in the motor system, but to a more general dysfunction of the neural network involved in the organization of voluntary action. In agreement with this, in a recent Functional magnetic resonance imaging (fMRI) study Bartolo et al. (2009) showed that the neural network involved in reachability judgments encompasses a fronto-parietal circuit including the cerebellum, similar to the one subtending actual motor action (Jeannerod, 2006). Patients with RH damage might thus suffer from a specific deficit affecting the organization of voluntary motor action in response to the environmental stimuli, which was identifiable when using either arm. However, our results also showed that reachability judgments are more affected when performed in relation to the hemiplegic than the healthy arm in RH and LH groups. This may indicate that the motor system responsible for hemiplegia has nevertheless a determinant role within this network. In agreement with this, LH patients did not show such abnormal performances when considering the healthy hand, and showed performances in the reachability and motor imagery tasks similar to the control group.

According to the literature, the LH plays an important role in the control of complex motor skills and trajectory execution, whereas the planning of voluntary action relies predominantly on the RH. In particular, right-handed patients with lesions in the hemisphere controlling the non-dominant arm was found to produce mainly deficits in the accuracy of the final position of the dominant arm, suggesting a specific impairment in accurately planning voluntary motor action (Haaland and Delaney, 1981; Winstein and Pohl, 1995; Haaland and Harrington, 1996; Prestopnik et al., 2003). By contrast, lesions in the hemisphere controlling the dominant arm was found to mainly produce deficits in the spatio-temporal features of motor trajectories, suggesting a deficit in the on-line control of voluntary action (Haaland and Delaney, 1981; Haaland and Harrington, 1996; Prestopnik et al., 2003). Our results are in agreement with this distribution of function between the right and LHs. Indeed, the deficits in judging what is reachable, a perceptual task involving the motor system, were essentially observed in the patients belonging to the RH group.

Moreover, impairment in planning voluntary motor action seems to correlate with the difficulty in imagining the same motor action. Indeed, a wealth of data have shown overlapping neural networks in actual and imagined motor tasks (Dominey et al., 1995; Sirigu et al., 1995) though some differences have also been noted (Gerardin et al., 2000; Kroliczak et al., 2007). Motor imagery is thus thought to involve motor planning processes (Jeannerod, 2001, 2006) and RH patients have been found to have impairments in planning actual motor actions and in motor imagery tasks (Stinear et al., 2007; Malouin et al., 2012). The motor imagery task we used involved complex motor planning (finger-to-thumb opposition task) and we therefore hypothesized a deficit in motor imagery in RH patients when compared to HC and LH groups. Results are consistent with this hypothesis. Indeed, participants with RH damage were statistically slower in the motor imagery task than the HC group and the patients with LH damage, when acting either with the healthy or the hemiplegic hand. Consequently, the slowness in the imagined motor task was concomitant with an increase in response times in the actual motor task. Furthermore, deficits in the motor imagery task in RH were associated with a broad deficit in the reachability judgment task, suggesting a possible link between the two tasks. This may be dependent on the fact that the motor imagery (finger-to-thumb opposition task) and the reachability judgment task involved both motor planning processes. Concerning LH group, we nonetheless observed slower response times in the imagined motor task compared to HC, but only for the hemiplegic hand. This finding confirms that motor imagery requires an intact motor neural network (Dominey et al., 1995; Sirigu et al., 1995). This also suggests that if the RH is predominantly dedicated to motor planning, motor imagery involved additional processing implying left motor regions when performed in reference to the right hand. In agreement with this, LH patients did not differ from the control participants in the reachability judgment task, which involved motor planning processes thought to be subtended by the RH, even when considering the hemiplegic hand. However, an alternative interpretation for the pattern of results observed in LH and RH could be that the performances in the motor imagery and reachability judgment tasks were not related, with a more detrimental effect of RH lesions on both tasks. This interpretation is however, not well supported by the observation in the reachability judgment task that RH patients, who showed broad impairments in the motor imagery task, are characterized by impaired response times only for stimuli located at the boundary of peripersonal space. This is indeed where motor-related information is expected to be highly determinant (see Coello et al., 2008). In support of this, we did not observe the same pattern of results in the color discrimination task. Hence, the key issues for the future would be to evaluate how brain insult resulting in hemiplegia relates to brain regions involved in motor planning, and whether those regions affect similarly actual motor performance, motor imagery as well as the perception of peripersonal space.

## CONCLUSION

In conclusion, the present study reports for the first time clinical data that argue in favor of a differential role of the left and right brain hemispheres in the perception of peripersonal space, in relation to the motor system. More specifically, our results highlight the role of the RH (in right-handed individuals) in motor planning and consequently, in motor-related perceptual and cognitive task.

## AUTHOR CONTRIBUTIONS

Angela Bartolo, Mauraine Carlier and Yann Coello collaborated in specifying the experimental design. Mauraine Carlier, Yves Martin and Sabrina Hassaini participated performing the neuropsychological investigations and collecting the data. Angela Bartolo, Mauraine Carlier and Yann Coello collaborated in the data analysis. Angela Bartolo, Mauraine Carlier, Sabrina Hassaini, Yves Martin and Yann Coello contributed to the writing of the article.

## Conflict of Interest Statement

The authors declare that the research was conducted in the absence of any commercial or financial relationships that could be construed as a potential conflict of interest.
